# The Clinical Implication of Cancer-Associated Microvasculature and Fibroblast in Advanced Colorectal Cancer Patients with Synchronous or Metachronous Metastases

**DOI:** 10.1371/journal.pone.0091811

**Published:** 2014-03-18

**Authors:** Yoonjin Kwak, Hee Eun Lee, Woo Ho Kim, Duck-Woo Kim, Sung-Bum Kang, Hye Seung Lee

**Affiliations:** 1 Department of Pathology, Seoul National University Hospital, Seoul, South Korea; 2 Department of Pathology, Seoul National University College of Medicine, Seoul, South Korea; 3 Department of Surgery, Seoul National University Bundang Hospital, Seongnam-si, Gyeonggi-do, South Korea; 4 Department of Pathology, Seoul National University Bundang Hospital, Seongnam-si, Gyeonggi-do, South Korea; The Chinese University of Hong Kong, Hong Kong

## Abstract

**Background:**

We aimed to evaluate the clinical significance of microvessel density (MVD), lymphatic vessel density (LVD), and cancer-associated fibroblasts (CAFs) in relation to tumor location in advanced colorectal cancer (CRC).

**Methods:**

Using immunohistochemistry, we examined 181 advanced CRC patients for CD31 and D2-40 to measure MVD and LVD, respectively, α-smooth muscle actin (SMA) and desmin to identify CAFs, and PTEN to examine genetic changes of CAFs. To evaluate the regional heterogeneity of these properties, we examined tissue from four sites (the center and periphery of the primary cancer, a distant metastasis, and a lymph node metastasis) in each patient.

**Results:**

MVD, LVD, and CAFs showed significant heterogeneity with respect to the tumor location. LVD was the greatest in the center of the primary cancers and the amount of CAFs was the lowest in distant metastases. In distant metastases, those from the lung had higher LVD and MVD, but fewer CAFs than those from the liver, peritoneum, or ovary. Patients with low MVD and LVD in the center of the primary cancer had worse outcomes and patients with few CAFs in distant metastases and in the primary tumor had a lower survival rate. PTEN expression in CAFs in distant metastases was lost in 11 of 181 CRC patients (6.1%), which was associated with a worse prognosis.

**Conclusions:**

The microenvironment, including cancer-associated microvasculature and fibroblasts, is heterogeneous with respect to the tumor location in CRC patients. Therefore, heterogeneity of microenvironments should be taken into account when managing CRC patients.

## Introduction

Although the mortality rates of colorectal cancer (CRC) patients have decreased in most western countries and in several developing countries in Asia, advanced CRC patients who initially present with stage IV disease or those who develop distant metastases several months after diagnosis still have a lower five-year survival rate [1], [2]._ENREF_4 Recently, the range of systemic chemotherapy has expanded and targeted therapy, including epidermal growth factor receptor (EGFR) and vascular endothelial growth factor (VEGF) inhibitor therapies, have been used in advanced CRC patients, increasing patient survival [3]. However, some CRC patients respond poorly to targeted therapy despite presenting positive results in targeted therapy-specific mutation studies [4]. One possible explanation for this therapeutic failure is tumor heterogeneity; several studies have reported that CRCs possess a heterogenic genotype or phenotype, including *KRAS*, p53, and *BRAF* [5]–[7]. Therefore, the differing characteristics of the primary tumor site and the corresponding metastatic organ need to be clarified to improve the management of CRC patients with metastatic diseases. Furthermore, understanding the clinicopathological characteristics of advanced CRC is important for the development and improvement of systemic therapies.

Since Paget et al. first described the cancer microenvironment by the “seed and soil” theory [8], there has been growing evidence that cancer-associated stroma might affect the cancer cells themselves and contribute to cancer progression [9]. The main components of the cancer microenvironment are microvasculature (microvessels and lymphatic vessels), inflammatory cells, and cancer-associated fibroblasts (CAFs) [10]–[12]. The current method of verifying angiogenetic and lymphangiogenetic activity in cancer tissue is to assess microvessel density (MVD) and lymphatic vessel density (LVD), respectively. MVD has been proposed as a surrogate marker of cancer-associated angiogenesis to identify patients with a high risk of recurrence or those with poor prognoses for various cancers including CRC [13], [14]; however, the prognostic correlation of angiogenesis in CRC is still controversial [15], [16]. Similar to angiogenesis, LVD has received interest as a means of lymphatic metastasis and survival [17], [18], but its role in tumor progression is still unclear [19]. The other prominent component of stroma, CAFs, are consistently activated and affect many aspects of tumor initiation, invasion, and progression [9]. While some studies have suggested that CAFs may inhibit tumor progression [20], [21], other studies have proposed that CAFs may promote progression in prostate, breast, and skin cancers [22]–[24]. In the context of CRC, Tsujino et al. have suggested that α-smooth muscle actin (SMA)-expressing CAFs might be a useful indicator of poor prognosis. However, these results were restricted to stage II and III CRCs [25].

In addition to cancer cells, genetic alterations in CAFs have demonstrated including the loss of heterozygosity, microsatellite instability, and genetic mutations [26], [27]. Recently, genetic inactivation of PTEN in CAFs was reported in breast cancer patients [28]. Trimboli et al. identified that PTEN loss in stromal fibroblasts resulted in extensive extracellular matrix remodeling and angiogenesis which characteristic of tumor progression [28]. However, expression loss of PTEN and its clinical significance have not been investigated in colorectal cancer patients.

The aim of this study was to investigate the characteristics of microenvironments, including microvasculatures and CAFs, in advanced CRC patients. Additionally, we assessed the intratumoral heterogeneity in the primary tumor and the discordance between primary tumor and distant metastasis microenvironments.

## Materials and Methods

### Patient selection

A total of 181 advanced CRC patients with synchronous or metachronous metastases, who were treated at Seoul National University Bundang Hospital (Seongnam-si, South Korea) between 2003 and 2009, were enrolled in this study. Synchronous metastases were defined as distant metastases occurring within six months of the primary diagnosis of CRC and metachronous metastases were those occurring after that time point [29]. The cancer tissue used in this study was received from patients that had surgical resection of both the primary tumor and related metastases. None of the patients had received chemo- or radiotherapy before the resection of the primary tumor. Medical charts and pathology reports were reviewed to record clinical and pathological data. Glass slides were reviewed to determine the histological type according to the WHO classification [30]. Follow-up information including the patient outcome and the time interval between the date of surgical resection and death was collected. The cases lost to follow-up and deaths from causes other than CRC were considered censored data for the survival analysis. The median follow-up period was 37.9 months (range, 0.8–104.6 months).

### Ethical statement

All human specimens were obtained from the files of surgically resected cases examined at the Department of Pathology, Seoul National University Bundang Hospital for the pathologic diagnosis. The retrospective study was performed using the stored samples after the pathologic diagnosis, and all of the samples were anonymized before the study. The participants did not provide written informed consent in this study. The study was approved by the Institutional Review Board of Seoul National University Bundang Hospital under the condition of anonymization (reference: B-1109/136-302).

### Tissue array methods

To evaluate the regional stromal differences, samples were taken from each patient from four areas: the center and periphery of the primary cancer, distant metastases, and lymph node metastases. The distant metastatic sites for the tissue arrays were as follows: liver in 83 cases (45.9%), lung in 38 cases (21.0%), seedings in 38 cases (21.0%), distant lymph nodes in 6 cases (3.3%), and ovary in 16 cases (8.3%). The representative core tissue specimens (2 mm in diameter) were taken from individual paraffin blocks and rearranged in new tissue array blocks using a trephine apparatus (Superbiochips Laboratories, Seoul, South Korea) [31].

### Immunohistochemistry

Array slides were labelled by immunohistochemistry using antibodies for CD31 (1∶100, DAKO, Glostrup, Denmark), D2-40 (1∶100, DAKO), SMA (1∶1000, Neomarkers, Fremont, CA, USA), desmin (1∶300, DAKO) and PTEN (1∶80, Epitomics, Burlingame, CA, USA) after a microwave antigen retrieval procedure except SMA. Non-reactive sites were blocked using 1% horse serum in Tris-buffered saline (pH 6.0) for 3 min. Primary antibodies were applied and antibody binding was detected with diaminobenzidine (DAB). Sections were counterstained with hematoxylin. The reactivity of PTEN in each tissue section was scored as negative, faint or strong, and the percentage of PTEN-positive fibroblasts was quantified. For the statistical analysis, the sample was deemed PTEN-positive if 5% or more CAFs were scored as strong positives.

### Calculation of LVD, MVD and CAFs using digital pathology

**Figure 1:**
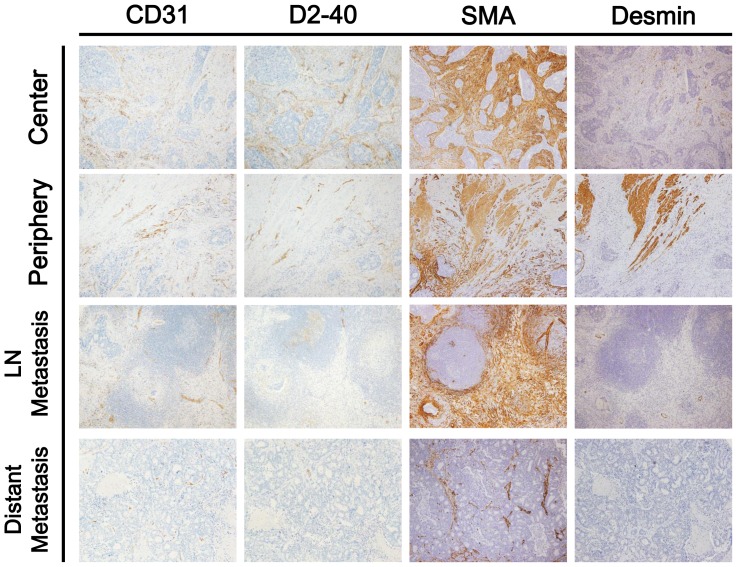
Representative sections from four tumor locations stained with CD31, D2-40, SMA or desmin antibodies (×100).

Slides were concurrently evaluated by two pathologists (H.E.L and H.S.L) using light microscopy to improve the accuracy of the results (Fig. 1). CRC cells were considered as internal negative controls. Medium- to large-sized vessels were considered as internal positive controls for CD31 and D2-40. Intestinal muscular layer or medium- to large-sized vessels were considered as internal positive controls for desmin and SMA. Samples showing inappropriate staining in internal negative or positive controls were considered non-informative and were excluded from the analysis. Slides were scanned using an Aperio ScanScope® CS instrument (Aperio Technologies, Inc., Vista, CA) at 20× magnification. Subsequently, they were analyzed in ImageScope™ using the Microvessel Analysis v1 algorithm (Aperio Technologies), and MVD and LVD were calculated. Because desmin-positive muscularis mucosa and propria are positive for SMA immunostaining, the area of CAFs (mm^2^) was calculated by subtracting the areas of desmin staining from that of SMA staining (SMA - desmin).

### Statistical analysis

A chi-squared test or Fisher's exact test (2-sided) for non-continuous variables and Mann-Whitney or Kruskal-Wallis analysis for continuous variables were used to compare each parameter with respect to the CRC site and according to its clinicopathologic features. The correlation between continuous variables was analyzed using the Pearson correlation coefficient. To determine the best cut-offs of continuous variables for predicting patient survival, the maximal chi-squared method was performed using R program (http://cran.r-project.org/). The overall survival curves were plotted using the Kaplan-Meier product-limit method and the significance of the differences between these curves was determined using the log-rank test. A univariate and multivariate regression analysis was performed using the Cox's proportional hazards model to determine hazard ratios (HRs). *P*-values of less than 0.05 were considered statistically significant. All statistical analysis, excluding the maximal chi-squared test, was performed with the IBM SPSS statistics 20 (Armonk, NY, USA).

## Results

### 1. Heterogeneity of cancer-associated stroma according to examined tumor locations

The clinicopathological characteristics of the advanced CRC patients are described in Table 1. The CRC patients with synchronous metastases had aggressive features including larger tumor size, more advanced pT and pN stage, and the presence of perineural and venous invasion than the patients with metachronous metastasis (p<0.05).

**Table 1:** Clinicopathologic characteristics of advanced colorectal cancers.

Parameters	Total	Metachronous	Synchronous	P value
	(n = 181)	(n = 57)	(n = 124)	
**Age (median, range)**	60.00 (28–93)	62.00 (36–79)	60.00 (28–93)	0.241
**Sex**				0.007
Male	97	39 (68.4%)	58 (46.8%)	
Female	84	18 (31.6%)	66 (53.2%)	
**Location**				0.055
Right colon	37	6 (10.5%)	31 (25.0%)	
Left colon	75	29 (50.9%)	46 (37.1%)	
Rectum	69	22 (38.6%)	47 (37.9%)	
**Size of primary tumor**	5.30 (2.0–13.0)	4.20 (2–9)	5.50 (2.5–13)	<0.001
**Histologic grade**				0.227
Low grade	157	52 (91.2%)	105 (84.7%)	
High grade	24	5 (8.8%)	19 (15.3%)	
**T stage**				<0.001
T1	0	0	0	
T2	5	3 (5.3%)	2 (1.6%)	
T3	107	45 (78.9%)	62 (50.0%)	
T4	69	9 (15.8%)	60 (48.4%)	
**N stage**				<0.001
N0	35	23 (40.4%)	12 (9.7%)	
N1	58	23 (40.4%)	35 (28.2%)	
N2	88	11 (19.3%)	77 (62.1%)	
**Perineural invasion**				0.011
Absent	89	36 (63.2%)	53 (42.7%)	
Present	92	21 (36.8%)	71 (57.3%)	
**Venous invasion**				0.028
Absent	126	46 (80.7%)	80 (64.5%)	
Present	55	11 (19.3%)	44 (35.5%)	

**Figure 2:**
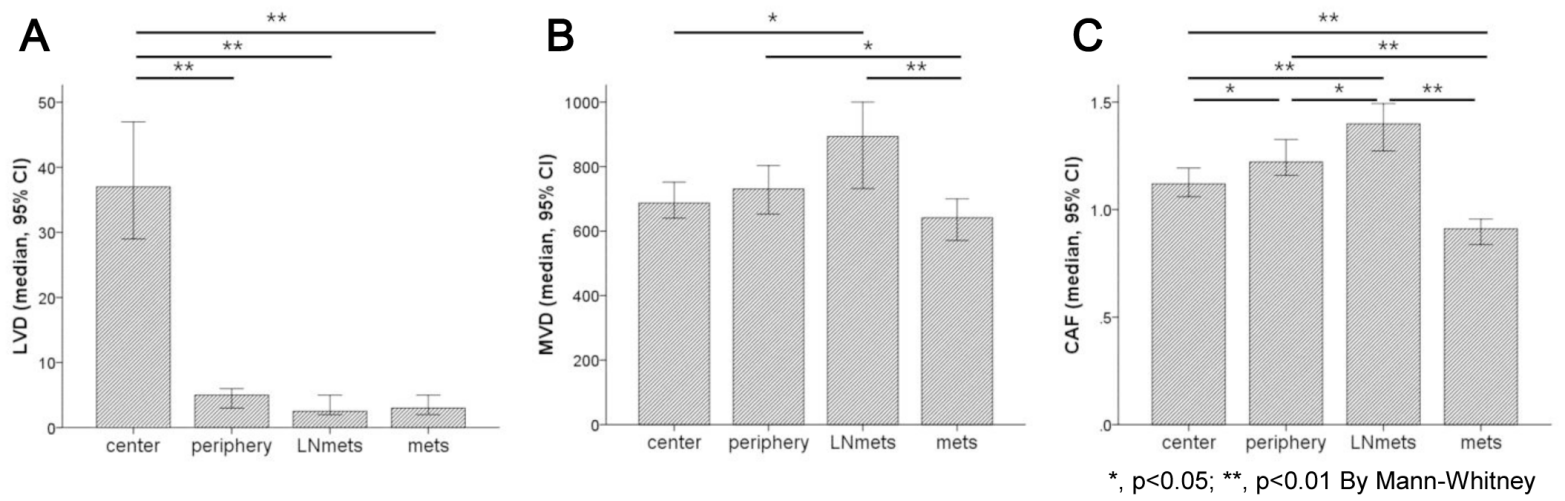
Heterogeneity of lymphatic vessel density (LVD), microvessel density (MVD), and amount of cancer-associated fibroblasts (CAFs) with respect to tumor location. The LVD (A), MVD (B) and CAF area (C) was significantly different according to each tumor location.

**Figure 3:**
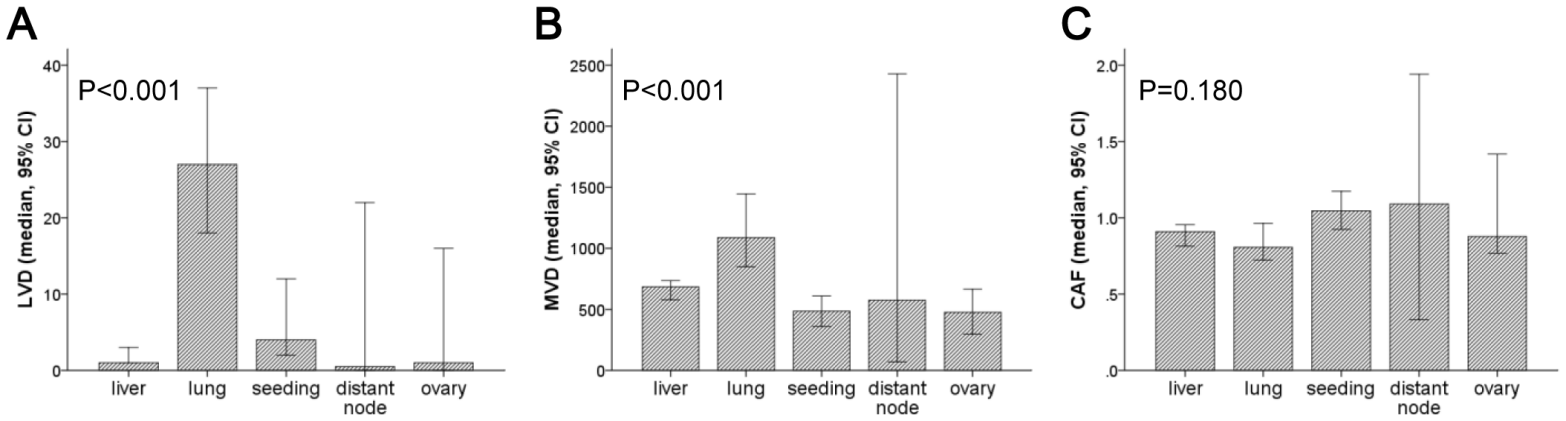
LVD, MVD, and CAF area at different distant metastasis sites. The characteristics of cancer-associated stroma differed with respect to the metastatic site. LVD (A) and MVD (B) were greater in the metastatic tumor samples collected from the lung than in samples collected from other metastatic sites (p<0.001). However, the amount of CAFs was not significant different between metastatic sites (C).

The heterogeneous values for LVD, MVD, and CAF area are shown in Fig 2. LVD was the highest in center of the primary cancers (median, interquartile range (IQR); 37.00, 10.50–81.00) than any other site (5.00, 1.00–23.75 at the periphery; 2.50, 1.00–15.00 in lymph node metastases; 3.00, 1.00–20.00 in distant metastases). MVD was lower in distant metastases (median, IQR; 641.50, 428.00–1006.75) than at the periphery of the primary cancer (731.00, 508.25–1049.75) and lymph node metastases (893.50, 520.25–1275.25). The area occupied by CAFs was the lowest in the distant metastases (median, IQR; 0.91, 0.68–1.18) than any other site (1.12, 0.88–1.41 in the center; 1.22, 0.96–1.54 in the periphery, 1.40, 1.00–1.71 in lymph node metastases). In addition, the stromal characteristics varied in relation to the metastatic organ examined. MVD and LVD were the higher in lung metastases than those in the liver, peritoneum or lymph nodes (p<0.001; Fig. 3). However, the amounts of CAFs were consistent among the different metastatic organs (p = 0.180).

Despite the heterogeneity of stromal characteristics, CRC cases with higher LVD, MVD and CAFs in center of the primary cancers had a tendency of higher LVD, MVD and CAFs in periphery (p<0.05; Table S1). However, LVD in center and periphery of primary cancer were not correlated with LVD in related distant metastasis (Table S1). In addition, the amount of microvasculature was significantly correlated with the amount of CAFs (Table S2).

### 2. Clinical significance of cancer-associated stroma in advanced CRCs

The MVD, LVD, and amount of CAFs present at each tumor location were compared according to their clinicopathologic features (Table 2). High grade CRCs were associated with lower CAFs in samples taken from the central cancer site (p = 0.041). When compared with synchronous metastases, the patients with metachronous metastases had higher LVD in center and periphery of the primary cancer and had higher MVD in lymph node metastases. Most patients with metachronous metastases were treated by adjuvant chemotherapy before metastasectomy. LVD and MVD in the distant metastases were significantly higher in the patients who had received chemotherapy before metastasectomy than those who did not (p = 0.011 and 0.048, respectively).

**Table 2:** Clinicopathologic factor and LVD, MVD and CAFs.

	Center (median)			Periphery (median)			LN metastasis (median)			Distant metastasis (median)		
	LVD	MVD	CAFs	LVD	MVD	CAFs	LVD	MVD	CAFs	LVD	MVD	CAFs
**Total**	39	717	1.13	5	740	1.22	3	888	1.42	3	648	0.91
**Histologic grade**												
Low grade	40	717	1.15*	5	741	1.23	3	895	1.43	3	665	0.92
High grade	34	683.5	0.94*	6	643.5	1.18	2	656	1.32	6	498	0.82
**pT stage**												
pT2	34	758	1.15	16	870	1.48				6	772	0.73
pT3	47	737	1.19	5	803	1.22	2.5	884	1.43	3	724	0.92
pT4	33	639	1.09	4	630	1.22	3	895	1.41	3	520	0.93
**LN metastasis**												
Absent	49	602	1.15	8	712	1.42				4	772	0.94
Present	39	737.5	1.12	4	740	1.21	3	884	1.41	3	617	0.91
**Perineural invasion**												
Absent	41	738	1.12	6	772	1.32	5.5	931.5	1.42	4	687	0.94
Present	39	672	1.13	4	702	1.2	2	796	1.39	3	548.5	0.86
**Metastasis**												
Synchronous	34*	717.5	1.11	3.0*	741	1.21	3	797*	1.39	3	617	0.93
Metachronous	55*	716	1.21	8.0*	712	1.23	2	1117*	1.63	5	698	0.91
**Chemotherapy†**												
Not done										2.0*	597.5*	0.93
Done										10.0*	684*	0.91

*, p<0.05; **, p<0.01; †, chemotherapy prior to metastatectomy of distant metastasis.

### 3. Expression loss of PTEN in CAFs

PTEN was expressed in cytoplasm and sometimes the nucleus of both cancer and non-neoplastic cells when examined using immunohistochemistry. Expression of PTEN was lost in 8 cases in the center, 2 cases in the periphery, 4 cases in lymph node metastases, and 11 cases in distant metastases (Table S3). In all 11 distant metastases with PTEN loss, PTEN expression was intact in both the center and periphery of primary cancer (data not shown). PTEN loss in distant metastasis was correlated with synchronous metastasis (p = 0.018).

### 4. Cancer-associated stroma and patient prognosis

By using the obtained cut-offs, lower LVD, MVD and CAFs in the center, LVD and CAFs in the periphery and MVD and CAFs in distant metastases were all significantly correlated with lower survival (p<0.05; Fig. S1). Among other clinicopathologic features, synchronous metastasis, old age, larger size, high histologic grade, advanced pT and pN stage and presence of perineural invasion were associated with a worse prognosis (Table 3). By multivariate Cox regression analysis, the hazard ratio of synchronous versus metachronous was the highest (4.029) with the lowest p value (p<0.001). CAFs in distant metastasis, LVD and MVD in the center, LVD in the periphery, age, and perineural invasion also independently predicted patient survival. In addition, loss of PTEN expression in CAFs in distant metastases was associated with a worse prognosis (p = 0.042; Fig S2), but not in primary cancer or lymph node metastasis.

**Table 3:** Univariate and multivariate survival analysis according to clinicopathologic features.

	Univariate survival analysis		Multivariate survival analysis	
Factors	HR (95% CI)	P value	HR (95% CI)	P value
Synchronous vs. Metachronous	4.617 (2.472–8.624)	<0.001	3.762 (1.838–7.701)	<0.001
Age	1.023 (1.004–1.044)	0.020	1.033 (1.011–1.056)	0.003
Sex (female vs. male)	1.428 (0.920–2.218)	0.113	—	—
Location (left vs. right)	0.503 (0.314–0.806)	0.004	0.700 (0.413–1.188)	NS (0.186)
Size	1.073 (1.005–1.146)	0.036	1.040 (0.903–1.198)	NS (0.584)
Histologic grade (high vs. low)	1.862 (1.061–3.269)	0.030	1.491 (0.763–2.912)	NS (0.243)
pT stage (pT4 vs. pT2/3)	2.341 (1.503–3.645)	<0.001	1.137 (0.674–1.921)	NS (0.630)
pN stage (pN1/2 vs. pN0)	3.848 (1.760–8.411)	0.001	1.773 (0.758–4.146)	NS (0.186)
Perineural invasion	2.628 (1.640–4.211)	<0.001	2.108 (1.265–3.513)	0.004
Venous invasion	1.217 (0.757–1.956)	0.418	—	
Center LVD (high vs. low)	0.364 (0.158–0.836)	0.017	0.298 (0.118–0.753)	0.010
Center MVD (high vs. low)	0.391 (0.233–0.655)	<0.001	0.437 (0.238–0.801)	0.007
Center CAFs (high vs. low)	0.579 (0.352–0.954)	0.032	1.038 (0.607–1.773)	NS (0.892)
Periphery LVD (high vs. low)	0.235 (0.086–0.644)	0.005	0.279 (0.096–0.809)	0.019
Periphery MVD (high vs. low)	1.456 (0.911–2.327)	0.117	—	
Periphery CAFs (high vs. low)	0.524 (0.336–0.817)	0.004	0.813 (0.499–1.326)	NS (0.406)
LN LVD (high vs. low)	1.646 (0.874–3.100)	0.123	—	
LN MVD (high vs. low)	0.597 (0.294–1.213)	0.154	—	
LN CAFs (high vs. low)	0.717 (0.423–1.217)	0.218	—	
Metastasis LVD (high vs. low)	0.569 (0.314–1.032)	0.063	—	
Metastasis MVD (high vs. low)	0.579 (0.364–0.921)	0.021	1.262 (0.720–2.211)	NS (0.417)
Metastasis CAFs (high vs. low)	0.492 (0.271–0.894)	0.020	0.290 (0.144–0.582)	0.001
Metastasis PTEN (intact vs. loss)	0.454 (0.208–0.993)	0.048	0.575 (0.239–1.383)	NS (0.217)

## Discussion

Carcinoma cells in different tissue areas have distinct characteristics [32]. In central areas of the tumor, carcinoma cells maintain an epithelial cell phenotype, but carcinoma cells in the invasive front acquire a more malignant and mesenchymal phenotype and are thought to have an increased migratory capacity and contribute to metastatic diseases. These metastatic cells may restore the epithelial phenotype at metastatic sites [33]. In addition to carcinoma cells themselves, microenvironment is suggested to be uneven within a given tumor because tumor formation and progression involve the co-evolution of cancer cells and microenvironments [34]. The present study demonstrated that the cancer-associated microenvironment also had distinct characteristics in different areas. Of the sites examined, LVD was highest in the center of the primary cancer. MVD was slightly higher in center than at the periphery of the primary cancer, but this difference was not statistically significant. Interestingly, the amount of CAFs in distant metastases was significantly lower than in center and periphery of the primary cancer. We show that the stromal microenvironment has regional heterogeneity both within the primary tumor and between the primary site and its related metastases. Furthermore, our data suggests that the stromal heterogeneity might be attributable to tumor heterogeneity. Therefore, it would be beneficial to consider both stromal and tumor cell heterogeneity in order to manage CRC patients better.

We evaluated the MVD, LVD, and amount of CAFs in metastatic tissues of various organs including the liver, lung, peritoneal seeding, distant lymph nodes, and ovary. Of the metastatic organs we examined, both LVD and MVD were the highest in lung. In our previous study, the *KRAS* discordance rate was also significantly higher in matched lung metastases than in other matched metastatic organs [35]. The underlying mechanism is not known. It could be that primary CRCs with high LVD and MVD have a tendency to produce lung metastases; however, our results indicated that LVD and MVD in the center and at the periphery of the primary cancers were lower in the patients with lung metastases (data not shown). Alternatively, it may be due to the physiological characteristics of metastatic organs, interactions between cancer cells and microenvironment within the metastatic organ, or the characteristics of the cancer cell clones prone to lung metastasis. However, technical or sampling errors also may be possible, thus further large-scale studies are required.

Although numerous studies have attempted to demonstrate an association between tumor microenvironment characteristics and survival, the prognostic impacts of MVD and LVD are still controversial. Some studies have been presented that active angiogenesis and lymphangiogenesis represented by high MVD and LVD are associated with poor prognosis and aggressive clinicopathologic factors [36], [37]. Recent meta-analysis has demonstrated that LVD was significantly associated with disease-free survival, but not overall survival [38]. Other studies have reported no statistical significance of MVD and LVD on survival [39]. Prall et al. has reported that high MVD and LVD are related with better survival in a consecutive series and liver metastases [40]. Our results were based on patients with advanced disease with distant metastasis and we showed that high MVD and LVD were related with improved survival. This might be because all the patients in this study had confirmed to have distant metastasis and microvasculatures could influence even delivery of the chemotherapeutic drug into the tumor. However, our study had some limitations in terms of the survival analysis. We enrolled the CRC patients with available surgically resected cancer tissues from both primary tumors and corresponding metastatic tumors. Not all advanced CRC patients with metastatic diseases were included and far advanced cases were not enrolled because of their inoperability. Therefore, unrecognized biases might have influenced our survival results.

Some studies have demonstrated an anti-tumorigenic effect of fibroblasts [20], [21]. However, it has become clear that CAFs contribute to the progression of cancer and their prognostic significance in various cancers also has been raised [41], and furthermore, several studies have observed genetic alterations in CAFs [26], [27]. PTEN loss of CAFs has been observed in breast cancer and prognostic association of it has been suggested [27], [28]. We observed PTEN loss of CAFs in CRC patients and it was more frequently observed in the corresponding distant metastases. It is suggested that CAFs, not only cancer cells, have altered gene expression. Moreover, loss of PTEN expression of CAFs in distant metastases was significantly correlated with the survival of patients. To our knowledge, these are the first results showing PTEN loss in CAFs in CRC patients. Although more research is required, we expect that it might be a prognostic factor in CRC patients.

In our large cohort of advanced CRC patients with synchronous and metachronous distant metastasis, we demonstrated the regional heterogeneity of stromal microenvironment factors according to the tumor location. The amount of microvasculature measured by LVD and MVD was also heterogeneous in relation to the metastatic organ examined. By Cox regression analysis, center LVD and MVD, periphery LVD, and CAFs in distant metastasis were independently associated with patients' prognosis in addition to synchronous distant metastasis, age, and perineural invasion. Heterogeneity of microenvironment, not only of cancer cells, is suggested to contribute to tumor heterogeneity and biologic complexity, thus it should be considered in managing CRC patients. In addition, our results showed that PTEN expression was altered in CAFs of CRCs, suggesting that CAFs might have altered gene expression and play an active role in cancer progression.

## Supporting Information

Figure S1 The prognostic association of stromal characteristics as it relates to tumor location. The analysis was performed by using cut-off values obtained by maximal chi-squared methods.
(TIF)

Figure S2 Representative PTEN antibody stainings of stromal cells and the prognostic association of PTEN expression. (A) Intact expression of PTEN in CAFs (×400) and (B) loss of PTEN expression in CAFs (×400). (C–F) Kaplan-Meier survival curves for the center (C) and periphery (D) of the primary tumor, lymph node metastases (E), and distant metastases (F) according to CAF PTEN expression status.
(TIF)

Table S1 Pearson's correlation coefficients among center, periphery, lymph node metastasis and distant metastasis.
(DOCX)

Table S2 Correlation coefficients between CAFs and LVD or MVD.
(DOCX)

Table S3 PTEN expression in CAFs and clinicopathologic factors.
(DOCX)
