# Supplementary figures and images for: The Clinical Implication of Cancer-Associated Microvasculature and Fibroblast in Advanced Colorectal Cancer Patients with Synchronous or Metachronous Metastases
Source: PLoS One. 2014 Mar 18;9(3):e91811. doi: 10.1371/journal.pone.0091811 (PMC3958375; doi:10.1371/journal.pone.0091811)

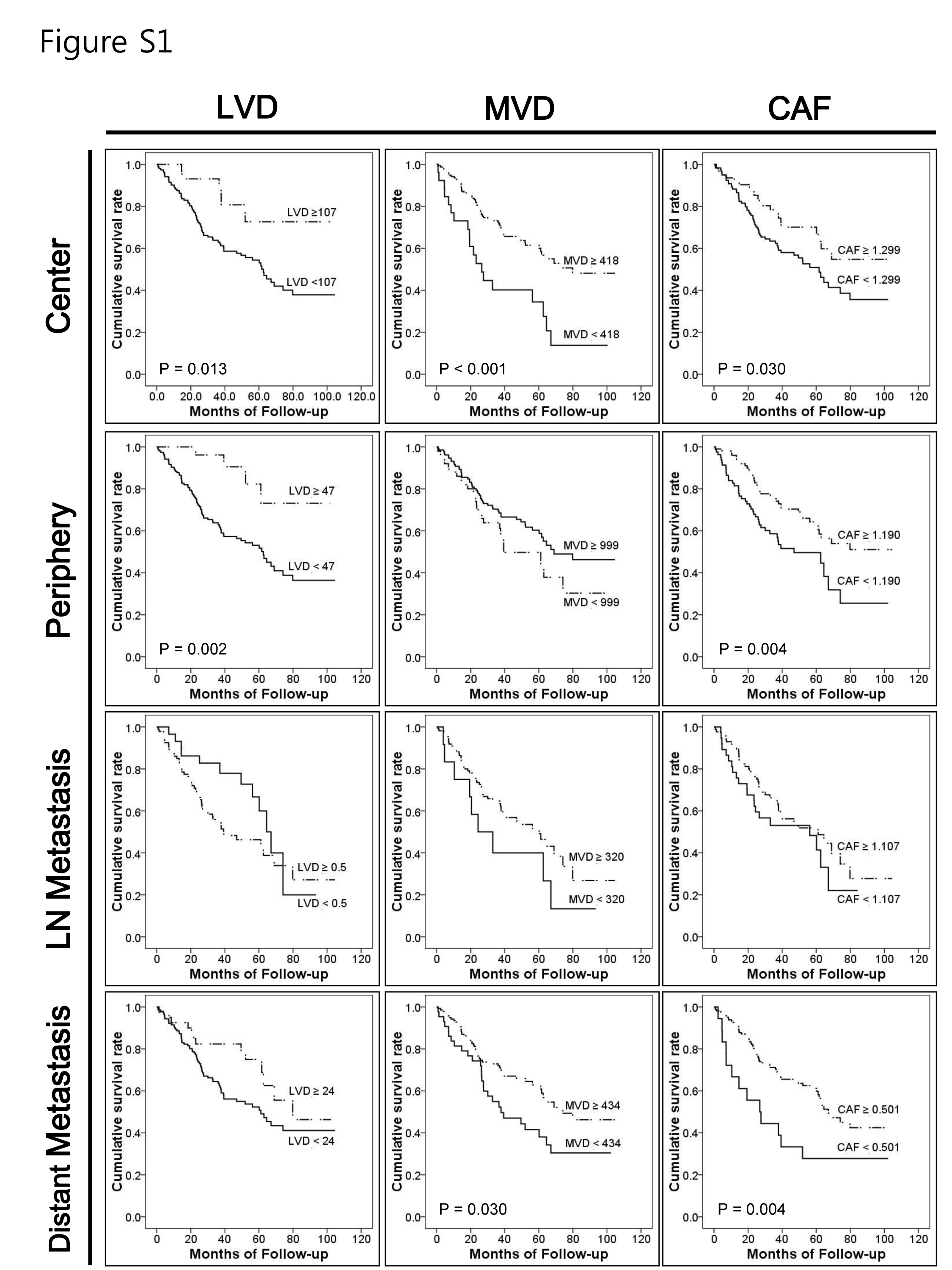

Supplement: Figure S1 — The prognostic association of stromal characteristics as it relates to tumor location. The analysis was performed by using cut-off values obtained by maximal chi-squared methods. (TIF) [file pone.0091811.s001.tif]

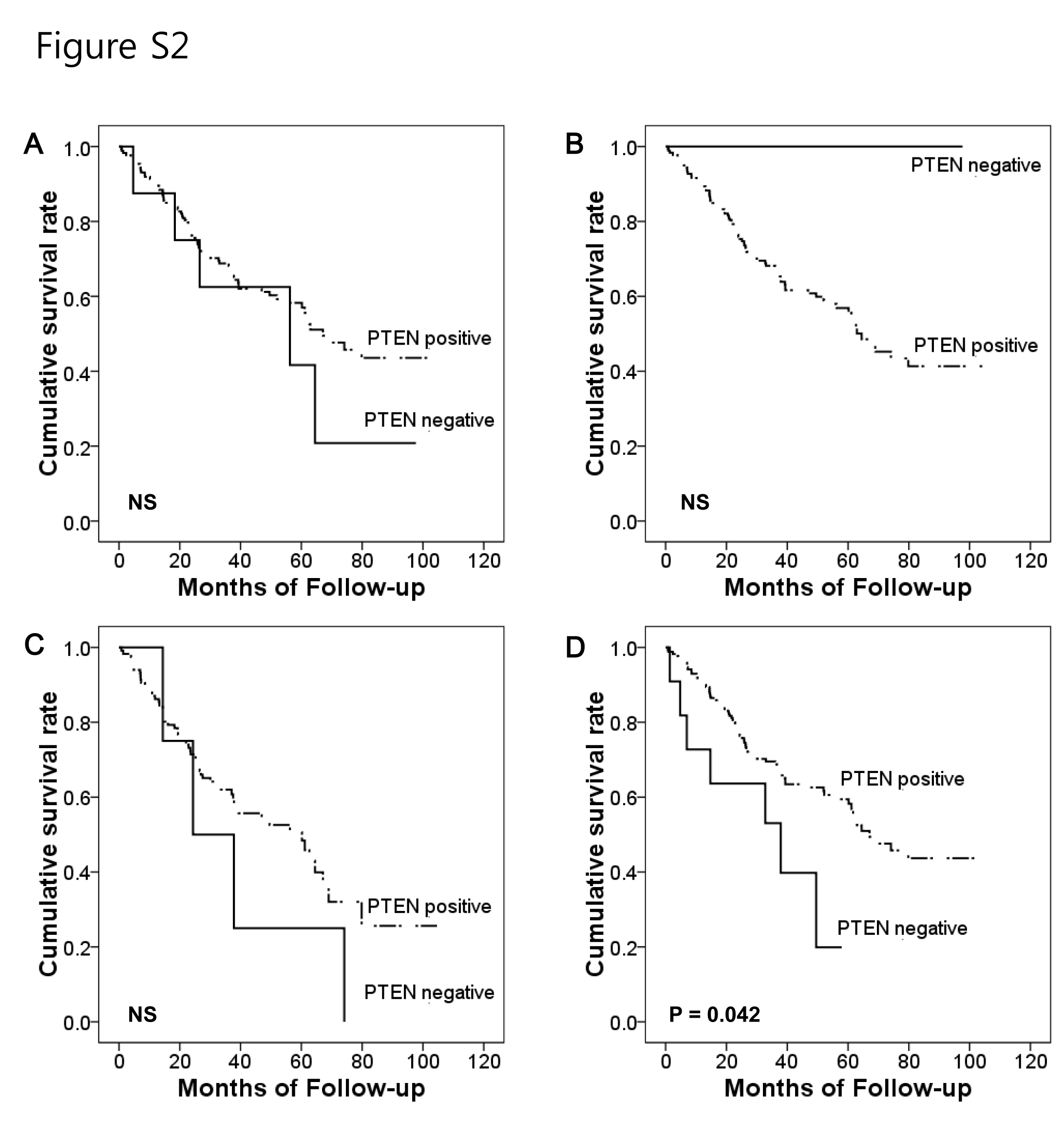

Supplement: Figure S2 — Representative PTEN antibody stainings of stromal cells and the prognostic association of PTEN expression. (A) Intact expression of PTEN in CAFs (×400) and (B) loss of PTEN expression in CAFs (×400). (C–F) Kaplan-Meier survival curves for the center (C) and periphery (D) of the primary tumor, lymph node metastases (E), and distant metastases (F) according to CAF PTEN expression status. (TIF) [file pone.0091811.s002.tif]
