# Supplementary material for: The Clinical Implication of Cancer-Associated Microvasculature and Fibroblast in Advanced Colorectal Cancer Patients with Synchronous or Metachronous Metastases
Source: PLoS One. 2014 Mar 18;9(3):e91811. doi: 10.1371/journal.pone.0091811 (PMC3958375; doi:10.1371/journal.pone.0091811)
Supplement: Table S1 — Pearson's correlation coefficients among center, periphery, lymph node metastasis and distant metastasis. (DOCX) [file pone.0091811.s003.docx]

Table S1. Pearson’s correlation coefficients among center, periphery, lymph node metastasis and distant metastasis

|  | LVD | MVD | CAFs |
| --- | --- | --- | --- |
| Center vs. periphery | 0.330** | 0.311** | 0.235** |
| Center vs. LNM | 0.308** | 0.255* | 0.226* |
| Center vs. DM | -0.034 | 0.147* | 0.165* |
| Periphery vs. LNM | 0.224* | 0.286** | 0.211* |
| Periphery vs. DM | 0.066 | 0.152* | 0.069 |
| LNM vs. DM | 0.095 | 0.093 | 0.095 |

*, p<0.05; **, p<0.01; LNM, lymph node metastasis; DM, distant metastasis
