# Supplementary material for: The Clinical Implication of Cancer-Associated Microvasculature and Fibroblast in Advanced Colorectal Cancer Patients with Synchronous or Metachronous Metastases
Source: PLoS One. 2014 Mar 18;9(3):e91811. doi: 10.1371/journal.pone.0091811 (PMC3958375; doi:10.1371/journal.pone.0091811)
Supplement: Table S2 — Correlation coefficients between CAFs and LVD or MVD. (DOCX) [file pone.0091811.s004.docx]

Table S2. Correlation coefficients between CAFs and LVD or MVD

|  | CAFs | | | |
| --- | --- | --- | --- | --- |
|  | Center | Periphery | Lymph node metastasis | Distant metastasis |
| LVD | 0.310** | 0.146* | 0.312** | 0.093 |
| MVD | 0.261** | 0.092 | 0.470** | 0.205** |

*, p<0.05; **, p<0.01
