# Supplementary material for: The Clinical Implication of Cancer-Associated Microvasculature and Fibroblast in Advanced Colorectal Cancer Patients with Synchronous or Metachronous Metastases
Source: PLoS One. 2014 Mar 18;9(3):e91811. doi: 10.1371/journal.pone.0091811 (PMC3958375; doi:10.1371/journal.pone.0091811)
Supplement: Table S3 — PTEN expression in CAFs and clinicopathologic factors. (DOCX) [file pone.0091811.s005.docx]

Table S3. PTEN expression in CAFs and clinicopathologic factors

|  |  | Total |  | Center | |  | Periphery | |  | Distant metastasis | |  | Total (LN mets) |  | LN metastasis | |
| --- | --- | --- | --- | --- | --- | --- | --- | --- | --- | --- | --- | --- | --- | --- | --- | --- |
|  |  |  |  | Loss of PTEN | P value |  | Loss of PTEN | P value |  | Loss of PTEN | P value |  |  |  | Loss of PTEN | P value |
| **Grade** |  |  |  |  | 0.287 |  |  | 1 |  |  | 1 |  |  |  |  | 0.503 |
| LG |  | 157 |  | 6 (3.8%) |  |  | 2 (1.3%) |  |  | 10 (6.4%) |  |  | 101 |  | 3 (3.0%) |  |
| HG |  | 24 |  | 2 (8.3%) |  |  | 0 (0%) |  |  | 1 (4.2%) |  |  | 19 |  | 1 (5.3%) |  |
| **pT** |  |  |  |  | 0.719 |  |  | 0.497 |  |  | 0.467 |  |  |  |  | 0.834 |
| pT2 |  | 5 |  | 0 (0%) |  |  | 0 (0%) |  |  | 0 (0%) |  |  | 1 |  | 0 (0%) |  |
| pT3 |  | 107 |  | 4 (3.7%) |  |  | 2 (1.9%) |  |  | 5 (4.7%) |  |  | 73 |  | 3 (4.1%) |  |
| pT4 |  | 69 |  | 4 (5.8%) |  |  | 0 (0%) |  |  | 6 (8.7%) |  |  | 46 |  | 1 (2.2%) |  |
| **LN mets** |  |  |  |  | 0.653 |  |  | 0.037 |  |  | 0.126 |  |  |  |  | 1 |
| Absent |  | 35 |  | 2 (5.7%) |  |  | 2 (5.7%) |  |  | 0 (0%) |  |  | 1 |  | 0 (0%) |  |
| Present |  | 146 |  | 6 (4.1%) |  |  | 0 (0%) |  |  | 11 (7.5%) |  |  | 119 |  | 4 (3.4%) |  |
| **LI** |  |  |  |  | 0.277 |  |  | 1 |  |  | 1 |  |  |  |  | 0.574 |
| Absent |  | 59 |  | 1 (1.7%) |  |  | 0 (0%) |  |  | 3 (5.1%) |  |  | 33 |  | 0 (0%) |  |
| Present |  | 122 |  | 7 (5.7%) |  |  | 2 (1.6%) |  |  | 8 (6.6%) |  |  | 87 |  | 4 (4.6%) |  |
| **VI** |  |  |  |  | 0.438 |  |  | 0.517 |  |  | 1 |  |  |  |  | 0.1 |
| Absent |  | 126 |  | 7 (5.6^) |  |  | 1 (0.8%) |  |  | 8 (6.3%) |  |  | 81 |  | 1 (1.2%) |  |
| Present |  | 55 |  | 1 (1.8%) |  |  | 1 (1.8%) |  |  | 3 (5.5%) |  |  | 39 |  | 3 (7.7%) |  |
| **PNI** |  |  |  |  | 0.279 |  |  | 1 |  |  | 0.381 |  |  |  |  | 0.632 |
| Absent |  | 89 |  | 2 (2.2%) |  |  | 1 (1.1%) |  |  | 4 (4.5%) |  |  | 52 |  | 1 (1.9%) |  |
| Present |  | 92 |  | 6 (6.5%) |  |  | 1 (1.1%) |  |  | 7 (7.6%) |  |  | 68 |  | 3 (4.4%) |  |
| **Border** |  |  |  |  | 0.507 |  |  | 1 |  |  | 0.603 |  |  |  |  | 0.216 |
| Expanding |  | 15 |  | 1 (6.7%) |  |  | 0 (0%) |  |  | 0 (0%) |  |  | 7 |  | 1 (14.3%) |  |
| Infiltrative |  | 166 |  | 7 (4.2%) |  |  | 2 (1.2%) |  |  | 11 (6.6%) |  |  | 113 |  | 3 (2.7%) |  |
| **Mets** |  |  |  |  | 0.708 |  |  | 0.098 |  |  | 0.018 |  |  |  |  | 1 |
| Synch |  | 124 |  | 5 (4.0%) |  |  | 0 (0%) |  |  | 11 (8.9%) |  |  | 91 |  | 3 (3.3%) |  |
| Metach |  | 57 |  | 3 (5.3%) |  |  | 2 (3.5%) |  |  | 0 (0%) |  |  | 29 |  | 1 (3.4%) |  |
| **Chemo** |  |  |  |  | 0.97 |  |  | 0.144 |  |  | 0.007 |  |  |  |  | 1 |
| Not done |  | 112 |  | 5 (4.5%) |  |  | 0 (0%) |  |  | 11 (9.8%) |  |  | 77 |  | 3 (3.9%) |  |
| done |  | 69 |  | 3 (4.3%) |  |  | 2 (2.9%) |  |  | 0 (0%) |  |  | 43 |  | 1 (2.3%) |  |
| Total |  | 181 |  | 8 (4.4%) |  |  | 2 (1.1%) |  |  | 11 (6.1%) |  |  | 120 |  | 4 (3.3%) |  |
